# Social Networking Sites and Perceived Content Influence: An Exploratory Analysis from Focus Groups with French Adolescents

**DOI:** 10.3390/ijerph17197025

**Published:** 2020-09-25

**Authors:** Boris Chapoton, Véronique Régnier Denois, Mabrouk Nekaa, Franck Chauvin, Valentin Flaudias

**Affiliations:** 1 Institut PRESAGE, Université Lyon, Université Saint-Etienne, HESPER EA 7425, F-42023 Saint-Etienne, France; veronique.regnier@univ-st-etienne.fr (V.R.D.); franck.chauvin@univ-st-etienne.fr (F.C.); 2 French Academic Directorate of the National Education Services of the Loire Department, Univ. Lyon, Université Saint-Etienne, HESPER EA 7425, F-69000 Lyon, France; mabrouk.nekaa@univ-st-etienne.fr; 3 CIRCEA, Université Clermont Auvergne, Université d’Auvergne, EA NPsy-Sydo, F-63000 Clermont-Ferrand, France; vflaudias@chu-clermontferrand.fr

**Keywords:** teenagers, influence, marketing, alcohol, tobacco, health literacy, behaviours

## Abstract

Social networking sites (SNSs) are invested in heavily by marketers aiming to reach a growing number of consumers. Concerns regarding the influence of posts displayed on SNSs in relation to behaviour were raised, in particular the promotion of ill-health behaviour directed to adolescents who may be at risk from suggestible practices. Although adolescents tend to be critical towards traditional forms of advertising, little is known about their perception of influencing strategies developed online, especially where sponsor- and user-generated content coexist. This exploratory study aims to gather information directly from adolescents about their use of SNSs their awareness of the influence that SNS content may have, particularly when it comes to tobacco and alcohol messages. Ten focus groups were conducted with 39 adolescents (11–16 years old; 56.4% male). Qualitative analysis documents the differences associated with adolescents’ favourite SNSs. The different parameters linked to each SNS and the expectation to find entertaining content and values associated with friendship may decrease adolescents’ perception of potential risk for health associated with SNS use. Authors advocate for the development of educational programs based on eHealth literacy and the use of social marketing techniques to facilitate and motivate adolescents to develop their competences.

## 1. Introduction

The influence of social media posts advertising alcohol and tobacco (A+T), in particular how they may influence the health behaviours of adolescent users of those sites, is an area of international research interest. Concerns regarding A+T posts displayed on social networking sites (SNSs) for example Snapchat, Facebook, Instagram, etc. have been researched internationally, particularly when it comes to their influence on adolescents’ behaviour towards these substances [1,2,3,4,5].

A+T are the two first avoidable risk factors of death in France [6,7]. Despite a decrease in overall tobacco consumption among the younger population (12–17 years old), French teenagers have one of the highest rates of use of A+T in Europe [8]. It is acknowledged that the teenage years are a period when the consumption of substances such as alcohol and tobacco may escalate from experimentation to misuse, and indeed to the development of habits that may lead to later addiction issues [9,10]. Research conducted by Health Behaviour in School-aged Children (HBSC) in 2010 and 2014 have documented that French teenagers commonly start to experiment with substance use and misuse (e.g., smoking and drinking) at the age of 13 years old [11,12]. By the age of 15, 61% of high school students have already tried smoking and 23% smoke on a daily basis [12]. Although the average age of the first intoxication remains stable at the age of 15, repeated alcohol use events (at least three times a year) and regular alcohol consumption (at least 10 times a year) have risen within the 17-year-old population [13].

One explanation may be the normative influence that teenagers could be under and the existence of persisting positive values associated with these products and their users [14]. Teenagers are very active on SNSs, making these social media platforms of particular interest when it comes to the study of such influences and the values given to specific behaviours related to health [15,16].

Marketing practices directed towards youth are denounced as affecting the perception of the need, the benefits and the risk of adopting the behaviour promoted [17]. In order to reach consumers, marketing strategies have evolved through the use of media (e.g., newspaper, TV, retailer), or through the message advertised (e.g., financial promotion, association to values, desirability) [18]. Although the quantity of exposure could be taken as an important variable to take into account when trying to link media influence to substance consumption [19], it is not the only variable of importance to consider. Several other qualitative variables must also be examined, e.g., the personal values shared through the ads [20] or the identification towards the people promoting the product [21].

Adolescents are particularly at risk of negative influence using such suggestible practices: the physical and psychological changes that occur through this period of life may increase their vulnerability and risk-taking behaviours, in particular because the information they receive may seem to be exaggerated, either positively or negatively [22,23]. Thus, adolescents can be influenced by the values associated with the use of the products advertised. This influence may result in the belief that use of such products may confer positive asocial benefits, for example being more popular or attractive or other valued social norms [5]. Adolescents tend to be critical over time towards regular forms of advertising and the common strategies used to target their interest [24]. Yet, the multiple forms of advertising strategies (e.g., traditional ad on billboard, product placement, sponsorship) are more difficult to avoid [25].

In 2007, it was estimated that an average American living in a city would see up to 5000 advertisements on a daily basis [26]. By 2015, this same exposure was estimated between 4000 and 10,000 advertisements daily [27]. Between these two dates, the use of the Internet has grown tremendously. The expectation is that there will be approximately 4.41 billion users of SNSs by 2025, a more than four-fold increase from 2010 user figures (0.97 billion) [28,29], leading to significant change in the landscape of advertising strategies. Indeed, social media platforms, on which the time spent by people far exceeds that spent on traditional media, allows producers, distributors, retailers and marketers to reach nearly half of the population worldwide [30]. These new opportunities to reach consumers and the economic issues at stake cannot be ignored: approximately 560 billion U.S. dollars were spent on advertising in 2019 [31], and about 50% of the spend on advertising is related to Internet ads [32]. The data generated by users of SNSs are valuable for companies as they allow marketers to get closer to the consumers by knowing their habits and preferences [33], resulting in the personalisation of adverts to fit the consumer’s lifestyle. Among the budget spent on advertising, a large amount of money is dedicated to a specific audience: adolescents [34]. Adolescents are an interesting target for companies because of their purchasing power integrated into the family shopping practice, and because of their individual purchasing power given by their pocket money. Moreover, once a preference for a brand has been developed in adolescents, this preference is more likely to last for a long time, generating multiple purchases over time [35].

Exposure to, and the influence of marketing strategies aimed at children, adolescents and young adults utilised in traditional forms of media are well documented [14,17]. The rising popularity of SNSs has promoted a shift away from traditional marketing strategies: new tools have been developed to reach audiences (e.g., display adverts, social media pages, celebrity/influencer endorsement) and have spread through different formats (e.g., video, photo, game, lens) while integrating audiences. Audiences have become part of the marketing strategies themselves by their interaction with, and creation of, personal media content associated with the product [3]. Consequently, different types of adverts exist within the SNS landscape: posts related to advertiser/marketer-generated content specifically aimed to reach consumers in a commercial strategy [36], and posts that are related to user-generated content created without any commercial marketing input (e.g., posting pictures with friends showing—deliberately or not—a specific brand/product) [1].

In this context, it is important for adolescents to have an awareness of the potential risk of content influence they may be exposed to while using SNS platforms. However, adolescents’ ability to make sound health decisions could be questioned [37]. Thus, the concept of eHealth literacy in this context is interesting to consider when appraising the need for adolescents to be protected against online influence that may impact their health. eHealth literacy is defined as “the ability to seek, find, understand, and appraise health information from electronic sources and apply the knowledge gained to addressing or solving a health problem” [38]. This concept has emerged in recent years and has become a priority within sustainable development goals worldwide [39,40,41]. In order to advance health literacy, the World Health Organisation (WHO) advocates that stakeholders include media and social media in the health literacy concept in order to “serve as a critical platform for health literacy messaging, harnessing the idealism and enthusiasm of youth, and meeting an ethical threshold for accuracy to support, rather than subvert, people’s right to health” [42]. Therefore, it is critical for researchers and policy makers to learn more about how adolescents use such SNSs. In order to empower adolescents and to provide them with a safe digital environment, researchers, practitioners and policy makers need to develop a precise knowledge of adolescents’ expectations and practices involving SNSs. They also need to be fully aware of potential advertising practices and susceptible strategies directed to adolescents, in order to act upon these. Recent research findings highlighting the causal relationship between alcohol marketing and youth drinking demonstrate the importance of this approach [43]. Furthermore, user-generated content could also promote the use of behaviours as much as, if not more than, the actual advertisements. Whereas restrictive laws have been legislated to protect people from online alcohol advertising, user-generated content could be hard to monitor and to regulate [44].

Although many researchers have tried to document the existence of a link between the use of SNSs and their impact on health over the last few years, these studies mainly focused on young adults over 18 years old, and used a quantitative approach, possibly leading adolescents into pre-established concepts. There is a dearth of research focusing on the voice of adolescents, particularly within the qualitative sphere. Moreover, to our knowledge, no studies on SNSs have been carried out focusing on French teenagers. This population could be of interest, as the French law restricts online A+T advertising directed at minors under 18.

Thus, the aim of this study was twofold. Firstly, it aimed to gather information directly from a sample of French adolescents about their preferences associated with SNSs. Important economic issues are associated with the use of one SNS over another. It is then important to regularly monitor the usage by adolescents of different SNSs and the differences perceived by adolescents between each, to ensure that specific potential risk can be identified. The second aim of the study was to explore if adolescents were paying attention to the influence that SNSs content could have on their behaviours. Particular attention was given to tobacco and alcohol posts displayed on their SNSs within this perspective. Sponsored posts associated with these products are not allowed in France when directed towards minors, however, this could be hard to monitor, particularly when independently generated by users outside of official commercial purpose.

## 2. Materials and Methods

This study was approved by the Institutional Review Boards of l’INSERM (IRB00003888, IORG0003254, FWA00005831) under the approval number 18-466. Parents signed and gave informed consent for themselves and for the children for whom they had authority. Children also gave written consent prior to participating in the research on a voluntary basis. Focus groups were conducted from February 2018 to April 2018.

### 2.1. Participants

Prior to the focus group starting, the researchers invited adolescents to fill a short paper questionnaire to gather socio-economic information (sex, age, family affluence scale (FAS III) [45]), and to request participation in a focus group to discuss their SNS use. Participation was possible on a voluntary basis, and no compensation was provided. The adolescents were in the French grade of 4ème (commonly 13–14 year olds) from two voluntary schools (one private school and one public school) or were participating in a community centre which caters for adolescents aged between 11 and 16 years old. This age group was targeted to fit the age range when substance consumption (A+T), as documented, begins to develop from experimentation to misuse [11,12,13].

Participants were assigned to the focus group by the educative team of each institution, according to the availability of the adolescents. Focus groups were considered by the researchers, as it involves interaction between participants: it allows participants to discuss themes collectively and to be challenged by alternative opinions [46]. Two conditions were given by the researchers to the voluntary schools and the community centre, in order to set up the focus groups. The first was to allow a minimum of 30 min for the adolescents to participate in the focus group and the second was to create groups of a minimum of two participants and a maximum of eight participants. The number of focus groups conducted was set in a convenient way, accordingly to the time available given by the institutions to the researchers.

### 2.2. Instrument

The focus group guide was semi-structured, developed and administered by trained researchers coming from a pluridisciplinary background (public health, psychology and sociology). The guide was developed in an exploratory perspective, using standard procedures [47,48] to gain insight into each of the SNSs that the teenagers were using regularly and to gather information about their awareness towards the possible influences SNSs could have on them, particularly when it came to A+T use.

A grounded theory approach was utilised, as it allowed for the researchers to learn from the participants who possessed the knowledge surrounding the phenomenon of interest [49]. The interview guide was arranged with general questions, not to suggest any response to the participants. The questions were divided in three sections within an exploratory perspective. The following domains were covered: the use of SNSs by participants, their perceptions of posts about A+T use and their perceptions of the risk of being influenced while using SNS. Throughout the focus groups, questions were adapted and specified to get a better understanding of SNS use by adolescents and to document their knowledge related to their use and the associated risks perceived.

Focus groups were recorded and transcribed professionally, independently from the research team. To ensure accuracy, transcripts were reviewed by the researcher, who led the focus groups to assess for any potential discrepancies.

### 2.3. Data Analysis

Data analysis was carried out in three steps, as described by Strauss, according to Couture [47]. The first step was achieved through open codification, which consisted of transcript analysis sentence-by-sentence, in order to identify relevant categories. Although the guide created was built upon three main sections that could be associated with independent categories, the researchers kept an open mind to the emergence of different categories. The second step consisted of creating an axial codification to go deeper into the significance of the categories. Axial codification follows four dimensions: (1) the conditions within which the categories have been established, (2) the consequences of each category, (3) the strategy used within each category and (4) the interactions between the categories. The third and final step used a selective codification, allowing the creation of links between each of the categories that emerged from the previous steps. Within this final step, only the main categories were considered, to avoid unnecessary noise. These categories had to be found on a regular basis within the transcripts, they had to be linked to other categories, they had to make a global theory possible, and they had to explain as much as possible the variation of the phenomenon.

The categories analysed and discussed below are those associated with the sections developed within the guide, and agreed among the pluridisciplinary team of researchers [50]. The results hereby summarised use a quote replete reporting method [51] and focus on the use of SNS by adolescents, followed by their perception of A+T substances and the risks they associate with SNS use. The data presented are based on the similarities of information that were gathered throughout the focus group, which allowed for conclusion to the point of data saturation within our sample [52].

## 3. Results

### 3.1. Population

As reported within Table 1, ten semi-structured focus groups were conducted with 39 adolescents: two focus groups with the community centre, six focus groups with the private school and two focus groups with the public school. The majority of respondents were male (56.4%). Focus groups lasted 45 min and 52 s on average (Standard Deviation (SD) ± 11:10).

Socio-economic data were analysed all together to preserve identity. Participants were 13.4 years old on average (SD ± 0.9); 36% of participants (*n* = 14) reported a low deprivation index (FAS III), 13% (*n* = 5) reported a high deprivation index, and 51% (*n* = 20) reported a medium deprivation index. Among the participants, 69% (*n* = 27) (69%) reported to have never used alcohol and 82% (*n* = 32) to have never smoked.

### 3.2. Definition of SNS According to Adolescents

The majority of adolescents have several SNS accounts, each with different features. These different features make it harder for adolescents to define precisely what an SNS is. No clear and unanimous definition is given to what an SNS is and confusion emerged when the adolescents took their own use of SNS as an example.
FG 8Boy 1 “*Well social networks, well, it’s a network where actually one is approachable to all, I mean to the world we can say. Well… well, it’s complicated, we can talk to several people, to many people…hum… well I don’t know…*” Boy 2 “*Let me do it! In fact, a social network is a place, a site where we will talk with buddies and post pictures, or not. And the purpose of a social network is to communicate. It’s open to adults. We know that we shouldn’t have the right, you have to be 18 years old at least to be on… it depends which one…*”FG 1Girl 1 “*Snap and Insta. Twitter too it’s nice*” Girl 2 “*And Skype as well, Skype is nice. WhatsApp is good too*” Girl 1 “*Yes, but it’s not the same thing, you don’t understand*” Girl 2 “*But it’s networks as well*”FG 6Girl “*Well it’s more for girls; there is Musical.ly (Currently named Tik Tok). But, me, I do not really consider it as a social network, I mean it’s video in playback and, me, I don’t see it as a social network really-really, like first there is not a lot of people…*” Boy “*It’s starting to…*” Girl “*Yes it’s starting to, but it’s more in America. We can do live video but we have to download another application, and you can send messages, but it’s not the same, well I think […]*” Boy “*It’s not enough… It’s not something that… it’s not a social network. I couldn’t say that Musical.ly is a social network site because it’s more about entertaining. You really have to do playback on a music, you don’t communicate with others*”

Most adolescents see SNSs as a way to share moments of their lives with people close or far from them and with whom they share a common interest. They use SNSs to have the possibility to be in contact with these people. These contacts could be made between two persons or within a group of persons. SNSs give the opportunity to adolescents to experiment with the world and to share their own experience of it. Adolescents use SNSs to be informed about the news that people are discussing, new events and new products that exist, but above all, they use SNSs to keep in contact with people.
FG 2Boy 1 and Boy 2 [at the same time] “*It’s a network to communicate*” Girl 1 “*And to get information. Well it’s made to communicate with my friends…*” Boy 1 “*It’s to know what is happening in the world*” Girl 1 “*Yeah and to be informed about life*”FG 6Boy “*Social networks, it allows to share, what you do, for example when you go on holidays, or to share nice pictures of you or even a landscape, to talk with people, through private message for example, that’s it. I mean, after people can also comment about what we have done, on what… for example, on Instagram, it’s the one that I use the most, it’s… you can post pictures, people can like your picture and after they comment. If they find that your pictures are nice, they can fallow your Instagram account and that’s it*” FG 2Boy “*With Social Networks Sites we know about life, what’s going on around us, like the death of Johnny (Johnny Hallyday, a famous French artist), I heard about it on Instagram*”FG 3Girl “*It’s to talk about what you do in your personal life. It’s to show what you like*.”FG 4Boy “*The thing with social network sites is that, contrary to mobile phone, we can create group and talk within a group. I think it’s the reason why we use Snapchat and Instagram*”

### 3.3. A Variety of SNSs for Different Uses

All of the adolescents from the focus groups declare to have an account on at least one SNS and to have different uses associated with them.

When asking the adolescents to cite the SNSs they know, some SNSs are recurrently cited by the groups of adolescents whereas other would be more user-specific. Snapchat and Instagram are the application most cited (by each of the 10 focus groups), followed by Twitter (cited by seven focus groups), Facebook and YouTube (cited by six focus groups), and then Discord, Musical.ly (currently TikTok) and WhatsApp (cited by two focus groups). One focus group also cited Skype, Tinder and Messenger as SNSs they knew.

Although some focus group participants did not cite YouTube, WhatsApp or Messenger when asked SNSs they knew, these applications could have been mentioned by the participants during the interviews while discussing their use of SNS, underlying the blurred lines between what an SNS is and what it is not.

On one hand, YouTube is described as being mainly used to watch entertaining videos to relax. Specific videos would be researched by adolescents that are involved within the gaming world or when they want to know more information about a specific subject. On the other hand, WhatsApp and Messenger are used mainly for group discussion within a family context and/or when participants live apart from each other in different countries.
FG 2Boy 1 “*For me, on YouTube I mainly watch gamin*” […] Boy 2 “*The thing is that on YouTube you find most of the time what you are looking for. Thus, we often have the solution. For me, it is quite helpful for my studies*”FG 10Boy 1 “*On the text messaging system you can only discuss in France, whereas on WhatsApp, you can talk to other country*” Boy 2 “*You can talk to other country, but you have to pay*” Boy 1” *On WhatsApp you can, it’s free*”

The type of SNS used by adolescents is partly justified as a fashion trend. The historic SNS Facebook is seen as old fashioned, used mainly by older people over 30. Facebook is also mentioned as a means to get in touch with people that could have disappeared from one’s life at some stage. Adolescents do not see any interest for them in using this application as they are living in the present. They are mainly interested in communicating with their peers; as such, they would not use Facebook to do this, since their peers would not have an account on this social platform or the account would not be active. Some of the adolescents have created a Facebook account for a specific purpose (e.g., to earn a reward within a game or to facilitate the access to some websites), whereas others may have a Facebook account, but do not use it anymore, or have deleted it.
FG 4Boy 1 “*Me I have a Facebook account, but it’s only for games, it’s to earn some…*” Boy 2 “*Yes me too!*” Boy 1 “*It allows you to move forward within the game so it’s cool*” […] Boy 3 “*Yes, me it’s the same, as for an example in some games there are some rewards or simply because we can’t access a game if we don’t have any Facebook account. So we use it*” Boy 1 “*We create fake account*”

Most of the adolescents interviewed had an active account on different SNSs and were even using two different SNSs to communicate with the same person.
FG 4Boy 1 “*So on Instagram, it’s the same it’s to talk with friends. Twitter it’s more to share what I like and… that’s it. And Discord is a thing for video games, to talk with other people that play at video games*” Boy 2 “*Yes that’s it, Discord is more to meet people that we don’t know about and to play video games*” Boy 3 “*Yes, no, me it’s… I have it too but, me as well I… to exchange advice*”FG 2Boy “*Facebook, like it’s more for a private network, like my family and so on. It’s more like a social network for old people… if I can say so. After you have snapchat that is more… I don’t even know all my contact there. Instagram, it’s all my friends and Twitter it’s a bit… I look at what is going on in the world. It’s a bit like Google news*” Girl “*Me, it’s not the same, Facebook it’s to look at the news and on. And actually, on Facebook there is more people that I don’t know than on Snap. And on Snap, I almost know all of them. And then, on Instagram, it’s more to look at celebrities, their account and on.*”FG 1Boy 1 “*There is no favourite but… I don’t know, honestly, I don’t know, we can’t compare them, it’s not the same thing*” Girl 1 “*We can’t compare. I don’t have a favourite*” Boy 1 “*It’s the same thing, not exactly, but it’s…*” Girl 2 “*At the end, it’s kind of the same things, you speak with the same persons or not?*” Boy 1 “*Yes, it’s true*” Girl 2 “*I don’t know, but for me, I can talk on two SNSs with the same person*” Boy 1 “*Yes, me too, exactly*” Girl 2 “*But I won’t talk about the same thing*” [Group agree]

### 3.4. SNSs are Associated with Communication and Friendship

Adolescents are engaged within different types of discussion and with different people. These discussions could be between two individuals, or group discussions. It would appear that the groups of friends that are known in person by the adolescents are transposed into SNSs groups, which are created specifically for these known people by the adolescents.

Adolescents mention having groups associated with their leisure activities, with their class from school to discuss homework, or having groups associated with the people they spend time with at school (depending on their acquaintance), etc. They would not share the same content with each of the groups they have created. Adolescents expect, as well, to find differences with what is shared within each group and with people: some girls report not discussing the same things with a girls-only group as with a mixed-gender-group. They do not even expect boys-only groups to discuss the same subject of interest as them.
FG 3Girl 1 “*It’s boys so I think that it’s not the same because for example, girls, well not all the girls, but, well me, I really love to take pictures, whereas boys it’s not the same I think. It’s not… no I think that the use of social network sites wouldn’t be the same at all*” […] Girl 2 “*It depends because with girls it… we do not particularly talk about the same things, I mean, I don’t know how to say it, but I know that I have a small group of friends where there are girls and boys, but I know that it’s real friends. Then, I mean I can say anything to them. But after If I want to say something… personal, well I can say it to… I mean I say it to the girls only.*”

Adolescents acknowledge that their two most used SNSs, Instagram and Snapchat, are getting similar to one another over time when it comes to the services they offer to their users. They appreciate these applications, because of the possibility to send photos and videos to communicate, and not only written messages.
FG 10Boy “*Sometimes I post a picture just like that, to ask about homework for example. It’s just that I don’t want to write and thus, well I talk and post a video. Otherwise, if it’s about a funny topic or I don’t know, and for example we are in a group with several friends and we try, I don’t know, it’s a joke, well it’s funny then, so we try to make jokes and thus it avoids us to write them down because at least there is the mimics of the face when we say it*”

One function of Snapchat compared to Instagram is the possibility to create a Snapstreak with another contact. A Snapstreak with one or many individuals is symbolized with the creation of a fire/a flame emoji that appears next to the profile of the contact with who snaps are sent back to each other every day more than three days in a row. The occurrence of this symbol of friendship may have different connotations. As this flame elevates one’s friendship, it can create some new friendships and it can nudge users to stay in contact with their friends in a positive way. On the other side, the loss of this symbol of friendship, which could happen when no snap has been sent during 24 h, could be hurtful for the ones that invested themselves into its existence.
FG 10Boy “*For the picture, for example, I create flames, it will be about many days, I mean it’s really a kind of number of days that we talk with someone and we can send pictures. I mean we have to send pictures but not necessarily… well when you say we share pictures, for me, it’s not pictures of me. For example, I don’t know, I put my hand in front of the flower vase and it’s good I get a small emoji of a flame and at least people, they know it’s the picture of the day and that’s it. And it’s true. Then they don’t like… no… no pictures of me every day then, I’m not gonna… […] After I mean it’s not that important, I mean… for example, even someone who had a full year sending pictures with somebody, for sure it will be a little annoying, for a little while that he has lost it, but after all it’s not vital. We can have flames, well we start over again, and I don’t think it is that important. For some, it’s all their life, I mean if they lose 3 of them they are like “woooh”, I mean it’s true*” FG 4Boy 1 “*On Snapchat there is a thing, the fire system, I don’t have the application myself, but what is it exactly? Because it looks like to be quite addictive that thing. Yeah I think that it’s also a problem with Snapchat, it’s… intrusive. It’s… it’s an incentive to use the application, I mean that’s what I think. […] after three days, there is the fire for example and the idea is to go as far as possible, 100 fires, things like that. And then, yeah, there is not really… connection because, like… for example, if someone create a fire with 10 persons, then she will take a picture… 10 persons it’s not really, I mean there is no connection, talking, things like that*” Boy 2 “*Well yes actually, you can stay in touch with people that you don’t see, far from…*” Boy 1 “*Yes, but if you take a picture of the table it’s not…*” Boy 2 “*Well like that it makes you stay in touch with people. I mean more or less but… if they are at the bottom of your list, you don’t think about it. Whereas if you see them at the top, you could think about sending them a message sometimes or things like that*”.FG 1Girl 1 “*I think that when you lose your fires, it’s a grieving. (Group agree) Losing fires is horrible, you lose 170 just like that, pff*” […] Girl 2 “*I don’t know how to explain; it depends relationships, I don’t know, it’s strange, first it allows to… well, fires are useless, we can agree on that (lough) but here we are.*” Girl 1 “*For me it’s a grieving when I lose my fires, I’m like “oh no, I’ve lost my fires, it’s a joke*” Group “*Yet it’s useless*” Girl 1 “*Yet it’s just useless*” Girl 2 “*It’s useless, but it allows you to stay in touch etc., that is good. That’s the word I was looking for*”

Although adolescents consider having only friends as a contact that they are communicating with, it seems that the definition of friends needs to be taken in a broad way. Adolescents seem to get in touch with a wide variety of people, starting with their closest friends, but also with the friends of their friends. Adolescents are also connected to people they have met on particular occasions, to people they can regularly see without necessarily having talked to them (e.g., older people from school), or to people with whom they seem to share common interests, and that they got to know through the SNS itself (recommended by the SNS itself, or when they have received a friend request).

While some participants mention that they would not particularly contact or follow people that are not their age, this affirmation is tempered by who the older person is. These adolescents would follow people that are older than them, such as teachers or physiotherapists without necessarily talking to them, rather just to acknowledge their acquaintance. They do not mention applying specific settings to these people in order to control what these people can see from their profile or their posts. Still, when it comes to family members, adolescents control and reduce the access of family members to their profile or their posts, especially for their parents.

Cousins or aunts tend to have the same access as the adolescents’ friends to the content posted. The majority of adolescents do not have their parents as a contact on their SNS they use the most. When connected with their parents on SNS, adolescents control some of the content widely shared, so that they would not be annoyed by comments from their parents about their private life or about behaviours that they know are not valued (e.g., answering to insults, smoking, love life, etc.).

### 3.5. The Privacy Associated to SNS

Whereas some adolescents know precisely how to apply the privacy settings to the posts they do not want to share with anyone (or with specific people only, like their parents), some adolescents do not know how to set up these parameters on a private mode.

Some adolescents raise concerns about the personal information they share through their account on SNS and their posts. Indeed, automatically as a default mode, profiles created on the favourite SNSs of adolescents are set to be public, so the information associated with the user and its account can be shared and seen by everybody. If adolescents want to control the information they share about themselves while sharing a post, their privacy settings need to be updated. Yet, the way to secure their information is not clear for most of the adolescents and sometimes too complicated to worry about, as is reported.
FG 2Boy “*On Insta I’m on a private account. I have no choice but to accept people for them to see, but on Snap, well we can block the stories and all and you can block people but … [exhales] I couldn’t be bothered*”

However, the sense of privacy could be seen differently by adolescents. Some participants associate this privacy to the number of contacts they have on each SNS; others to the ability of people to see specifics of their life, such as the place where they live. Other adolescents associate this sense of privacy with a specific time or moment of their life, whereas for a few of the adolescents, life cannot remain private if you have an SNS account.
FG 1Girl “*I think that Snap is more private than Instagram […] On Insta I have about 700 followers and on Snap I have about 200 people… there is a difference you know, like, I think Snap it’s far more private.*”FG 5Boy 1 “*I have opened a Snapchat account and an Instagram account, but it didn’t interest me at all, so I deleted them*” Interviewer “*What did you not like about them?*” Boy 1 “*Well it’s really this way of… on Instagram it’s really about this type of showcase, we post picture of ourselves… Personally, I don’t really want that people look at my life, I want to…. I want to keep it for myself actually. And Snapchat, well, I don’t really see any interest in posting pictures to everybody all the time since…. There are messages for that, if I want to communicate, with messages I can. No need to… send pictures*” Girl “*Yeah, he is not wrong in a way*” Boy 2 “*Yes, he is right*”

The privacy of the information shared on SNSs and the risks associated with the person who can access the information were questioned by some of the adolescents. Whereas most of the adolescents will tend to put into perspective these risks, few do not want to lose control over the information they are posting. Other adolescents associate privacy with the possibility of preventing some undesirable people to contact them or with the ease to find and access their profile. Some adolescents shared their experiences regarding this perspective by explaining the way of blocking someone from an SNS. Snapchat is considered as being more protective towards one’s privacy. This is explained by the need to know the alias of the person one wants to add prior to doing it. The avatar used on the SNS’s profile is as well created from a Bitmoji (A Bitmoji is a personalized cartoon avatar), rather than from a picture, which could make it harder to identify precisely the person behind this avatar. Moreover, if one wants to block a contact, it can be done easily, without fearing being reached through another way (e.g., by calling or sending text messages), since the phone number would not be shared and would not be needed in the first place to add a contact.
FG 1Girl 1 “*I think that Snapchat is much more private than Instagram*” Boy 1 “*Yes, I also think it is. For Snap, we can choose the person and on Instagram, you see, like the person you have in common, so like Snapchat, but it’s like… Snapchat is more researched, you look more for the person, but in Insta, it’s not…*” Boy 2 “*And you can talk more on Snapchat than on Instagram, like through messages*” Girl 2 “*Also I think it’s better than giving your phone number, directly, you can block the person if she bothers you too much, I mean between brackets*” Boy 1 “*Oh yes, that is what I often do*”

Although adolescents mention the danger of sharing private information with people, they also acknowledge the possibility for them to use SNSs in order to get to know someone better, with or without this other person knowing about it.
FG 1Girl 1 “*Well sometimes I do my little investigations, like with my buddies “wow look here, did you see one’s account” and on, you see. It’s like… I don’t know… we spy.*” Boy “*Yes, exactly. In private*”

### 3.6. Expectations and Specificities Given to SNS When Sharing a Post: Snapchat vs. Instagram

One post shared on an SNS would not be necessarily shared on another SNS account that the adolescents owned. This practice would be redundant for their contacts if they are following every account the adolescents have created. Thus, depending on the type of post shared, a single SNS would be used.

When sharing instant or live moments with their friends through SNSs, adolescents use Snapchat. This SNS is primarily about friendship and communicating with friends, as they would do face to face. Posts shared through this application are shared without giving too much thought about the quality of what is sent.
FG 2Boy 1 “*when I go to a football match, I post about the score*” Girl “*You post about your life. For example, I have a drink at a bar, a coffee, or I don’t know, I’ll take a picture and that’s it*” Boy 2 “*We post about the things that are a bit interesting, between quotes, what we did that is… it’s like to resume what we did, for example, sometimes with the divorced parents we are asked “oh what did you do today”: check my snap’s story*”

Whereas Snapchat is more considered by adolescents to send private posts with their friends, adolescents consider their Instagram profile as being accessible to a wider audience.

Consequently, they would build their profile with pictures that define who they are and what they like. The pictures posted on their Instagram account are selected in a more thoughtful way, as a kind of official profile that presents one’s interest, one’s passion and one’s life; all evolving over time. This profile could be used to enter other social platforms, such as YouTube, Discord and others. The pictures would be the ones found on their mobile phone, for example; they would be the ones saved, the ones to keep.
FG 5Boy 1 “*In fact it’s really different because Instagram it’s really to share picture of us, to share it on our account; whereas Snapchat it’s more like things that happen during the day. But it is not necessarily us that we post as a picture*” Girl “*And Instagram it’s for everybody that can see it, whereas Snapchat is for the ones that we accept*” Boy 1 “*Yes that’s it. Well Instagram can be in private as well but it’s not…*” Boy 2 “*Yes and you have less friends on Snapchat than on Instagram I think*” Boy 1 “*Yes, well it’s more people that you know*”FG 2Boy “*On Insta I post only picture a bit like normal, on Snap honestly I post sometimes stories a bit strange… I will not say more [laugh]*”FG 10Girl “*Sometimes I delete [my pictures], but they are old ones, but they annoy me, I don’t know. […] I was young, it was in the 6^th^ grade (“6^ème^” in France, equivalent of Year 7 (UK), 11–12 years old), I don’t know I don’t like it.*” Interviewer:
*it does not represent who you are anymore maybe, is that it?* Girl “*yeah, well I don’t like what I was posting, it was not… there was no quality, I mean it was ugly, we were seeing nothing that was nice*” 

This difference made between Snapchat and Instagram posts could be explained by the length of life of the post sent. Whereas a post sent on Instagram would last until the moment the person would delete it, a snap (photo or video taken through the Snapchat application. Once send and open by the addressee, the snap usually lasts a few seconds and disappear afterwards. The duration of life of a snap can be extended to 48 h or played twice, depending on the setting given by the sender.), or the message sent on Snapchat would disappear automatically within the next 24 or 48 h. Posts shared on Snapchat are not specifically made to engage the viewer into any interaction towards the posts. On the other hand, posts shared on Instagram allow people to comment on them or to like them. Adolescents do not expect much comment in relation to their posts, as the comments appear to be repetitive and related to the beauty of the post. If adolescents were expecting a reaction to their posts from someone, they would tag this person within the post shared. Rather than commenting on a post, adolescents give their appreciation of the post through a like. Likes are given automatically most of the time to the posts of the contacts they appreciate, without specifically weighting their true appreciation of the content of the post. Giving a like is a way of acknowledging that a post has been seen. As well, the likes given could also be an appreciation of the value associated with the relationship between the “poster” and the “viewer”.
FG 7Girl 1 “*When we know the person and we talk often, well we like and we comment. But when we don’t know the person, well, we do nothing. I mean, we like it.* Girl 2 “*Yes we usually only give a like*” Girl 1 “*There is that thing that, all the posts posted within a day or the previous days, they are all gathered. So, actually, we like, we scroll down, we like, we scroll down, we like. So… and then the people we don’t like, we don’t like*”. Girl 2 “ *Yes that’s it*” Interviewer “*And thus, is it the person that you don’t like or the picture?*” Girl 1 “*well it’s the pictures, since it’s the people we don’t like*”FG 8Boy “*It depends if he is close… mates near or far. I have some mates that I know… I know them but I don’t go there every time. I only like the picture.*” Girl “*Me I do not always comment.*” Boy “*Me I comment only the one that identify me, that’s it. These ones that are identifying you, it means that they put you on the picture, in a way. It means that if it’s your friends… and them, usually they should comment, it’s the least one can do. It’s a thank you.*” Interviewer “*So*” Girl “*I don’t know, me I like everything*” Boy “*The beauty. Or there are pictures that you only see their face, you don’t see any landscape, it won’t be great to look at. So if you see a picture of a guy at the beach, you see the outside and all. It will be prettier to watch.*” Girl “*No, me it’s if they are people that I don’t like, well I won’t give any like. I mean, I will give likes to people that I appreciate, but I think that it depends on the pictures, but honestly, it’s only for that*”. 

Adolescents do not report to have high expectations when posting on SNSs. They use the functionality that are given with the SNS if people interact with their posts, but none of the adolescents interviewed expected a major impact of their posts (e.g., becoming popular, being funded by a brand). Thus, when posting a story publicly (in the situation of a public profile) or restricted to all their contacts only (in the situation of a private profile), adolescents could look at the number of views they would get, but do not specifically wait for people to comment in relation to it; rather, to acknowledge that it has been seen.
FG 6Boy “*It depends on how many people are following you. Often, when you have more people who are following you, there is more people that will see your picture so they will like it more, but if for example we just start on some social networks sites, well we won’t have many followers, so not a lot of likes on the pictures, not many comments. Anyway, we post that, not really to… well it’s nice to have some reactions, but it’s to share, share nice moments.*” Girl 1 “*Me, I like even if I don’t like the picture*” Girl 2 “*Me it’s as if I was showing that I’ve seen the picture, like I don’t really care about the like, I like every picture and that’s it*”FG 4Boy “*It’s just to share, maybe a good time or things like that. But not necessarily any reactions. Even if there is the comments and the like, but I don’t think that there is really expectancies of reaction*”

The number of likes is not mentioned as being associated with a target. When someone wants to do it, it could be seen with the use of specific hashtags, or by posting pictures that are made to generate likes. One strategy to generate likes is to leave the content of their profile publicly available. Still, adolescents are able to differentiate between people that make a living from their posts and others.
FG 6Boy “*Either the person decides that she is doing that for fun, either the person decides that it will become her job. So she will make a living thanks to YouTube. […] On some social network sites as well, there are partnerships, for example on Instagram there are some. There are paid partnerships with this brand or that brand*” Girl “*For example, she wears… it’s a girl, she wears a brand sweatshirt, I don’t know, I say Levi’s randomly. Then she is under a paid partnership with Levi’s, so they give her clothes but in return she advertises for them*”FG 7Girl “*Snapchat, for me, it’s more to talk with friends, and to have a conversation, as if it was through messages. Instagram, it’s more… well it has become a job. It has become, like it’s all about influencer, blogger etc., well it’s increasing almost on Instagram. So Instagram it’s more for everybody to see, whereas Snapchat is more for our friends*”

Adolescents report a tendency to share positive posts most of the time, while acknowledging seeing less positive ones. Few adolescents report that posting on SNS is to make oneself look good rather than sharing and communicating with friends.
FG 2Boy 1 “*Me, my pictures are taken a bit on the fly. I want to post a picture on Instagram; I post a picture on Instagram*” Girl 1 “*Well not me!*” Boy 2 “*But there are some people to post about negative stuff*” Boy 1 “*Usually I post a blank picture, something simple*” Girl “*Yeah and the people that are more depressed, they would post a catchphrase picked on the Internet, negative…*” Interviewer: *Why do we not post negatives things?* Boy 1 “*Not to show that one has a rotten life*” Girl “*Me my life is not rotten*” Boy 1 “*I didn’t… no I don’t mean that, but there is a rotten side in life. Life is not all about happiness, teddy bears and pink things. We do not post ugly things, because people have enough in their life usually, well it depends who and also because it… it doesn’t show a good side of oneself*” Boy 2 “*And afterwards, people are like “well, look… he said that on his account”, I mean…*” Girl “*There a more judgement on the pictures that we post. People will judge depending on the appearance of the picture, of the comments we write on social network sites, or will say, “Oh this one, he put that because that’s the way he is in life; her, she post that picture because she is like that, and so on.*” 

### 3.7. Perception of Posts Associated to A+T Substances on SNS

When asking adolescents about the presence of alcohol- or tobacco-related substances on SNS, they do not recall at first seeing much if any from their friends. After a second thought and gradually from sometimes to always, they mention having seen posts related to A+T substances from some of their contacts.

Adolescents describe people who are sharing posts or stories that emphasise the use of A+T products negatively; however, they accept that it is normal to see such content on SNSs.
FG 6Boy “*I mean when drinking, it’s less shocking when for example you take a picture of a table because you have been … spending time with your family and there are two or three bottles of beer on the table, well it’s not shocking. We’ll not say “Oh well him, he drinks alcohol for that”. No we perfectly know that it’s not him, it’s a picture of a table where his family is present*”FG 8Boy “*It’s useless [to post about alcohol or tobacco]*” Girl 1 “*Well first, if it’s posted, it would be on Snap, because you would see it for a shorter length of time*” Boy: “*It’s to show off*” Girl 1 “*He is a seducer. Anyway everybody will or did it*” Boy “*No, I never did it. I have already smoked, but that never. I have never sent any picture, never*” Girl 1 ”*Well I did during an evening party, but only cocktails, that’s all*” Boy “*Dah cocktails, it’s alcohol*” Girl 2 “*Yeah but you really have to drink a lot of cocktail to get…*” FG 1Boy 1 “*talking about the presence of tobacco on post] “Some are doing it live, like John (Names provided within the verbatim are fictional) for example*” Girl 1 “*Peter is doing that continually. I see that on his Snap stories… “Yeah Shisha night”… ok sure do what you want but you don’t have to say it to everybody on the social networks, nobody cares*” Boy 2 “*Some are doing it to be cool really*” [Group agree] Boy 2 “*It’s to show off “yeah I smoke, I’m a grown up”.*”

When posting with alcohol, people, and especially their peers, are seen as trying to “show off” (FG2). Still, alcohol is trivialised, and adolescents consider it normal to see alcohol and to use alcohol during specific events. They do think that it is normal to drink alcohol especially when it is used by a person who is a bit older than them, or even when it is validated from their relatives, who may offer them a drink to celebrate a particular occasion.
FG 3Girl 1 “*Alcohol is… I mean alcohol for me, if you don’t drink much, all the time and so on, it’s not really bad*” Girl 2 “*but not at our age*” Girl 3 “*yeah, that’s it*” Girl 2 “*I think, well there are a lot that are doing it at our age. Because generations… I mean, I, personally, I notice that the generations… more… for example, before in 6th grade (Equivalent of 6^ème^ in France, 6th grade in United States of America and Germany, 7th grade in UK; approximatively 11 years old), there was nobody with mobile phone. Now, in 6th grade they all have an IPhone. And I think that’s too much. And thus they think they are grown up, so they think they can drink alcohol, smoke…*” Girl 1 “*There are people at school, when they invite each other’s, they drink, smoke shisha, they are… I mean… they do what they want, but not for me.”* Girl 3 “*They put that on Snapchat for example*” Interviewer “*Why do you say it’s too early? Is there eventually an age limit or…*” Girl 3 “*No but not our age*” Girl 2 “*Not an age limit but you shouldn’t…*” Girl 1 “*it should be over used*” Girl 2 “*that’s it*” Girl 1 “*With my family when there are birthdays, my parents they are like “oh you can have a small glass”, but that’s it. They are not like go ahead drink anything you want, no it’s just one glass. And if I don’t want any, they won’t oblige me to. My mother she says “if you don’t drink, I am really happy like that”… I mean… That’s it, a glass, for me it’s fine.*” Girl 3 “*Me I don’t drink, I don’t like that*” […] Girl 2 “*For example, I have celebrated the new year evening with my friends and there was other people who did it with their friends; well they had a lot of alcohol, whereas us we didn’t have any. It depends on the people, because for some it’s normal that at our age… yeah that’s it.*” Girl 1 “*they want to be the grown up*” […] Interviewer “*So if I understand it well, for you and today, you say that it’s not necessarily for you and you won’t necessarily drink much alcohol or when you have birthdays and on, but maybe that in two years times, maybe it would be more… normal?*” Girl 2 “*Yes*” Girl 1 “*Yeas that’s it*” Girl 3 “*I think that at the moment it’s not for our age*” FG 7Girl (talking about two profiles) “*Both of them had a bit of alcohol. I mean I think they are older than us. So, well, it’s totally normal*”

When asking about the association they make with alcohol, adolescents associate the substance to parties, students’ nights and nightclubs, as well as food and friends. On the negative side, alcohol is linked to intoxication and hangover. Few adolescents mention the addictive side of alcohol use, although this affirmation is tempered by an association made with depression. Most of the time, adolescents have integrated the use of alcohol as being normal, without any consequence.
FG 6Interviewer “*When we mention the term alcohol, what comes to your mind?*” Girl 1 & 2 “*me being intoxicated*” Boy “*Be drunk, because drinking alcohol from time to time is harmless*” Girl 1 “*personally, I’m scared, I don’t know, I don’t like it*” Girl 2 “*it’s to be used in moderation [laugh] until you don’t drink… I mean, I, I don’t drink alcohol, but I mean if adults, they are drunk once a year and on at a party, for a birthday, it’s not gonna kill them like*” Girl 1 “*For me, I don’t say I will never drink, but I don’t know, I’m afraid to get drunk [laugh]* Boy “*well you have to drink quite a lot to get drunk*”FG 7Girl 1 “*I have the image of the person who drinks, who… I mean who drinks normally, like two, three glasses, not much more. And then, I have the image of the guy who really drink a lot and yes… that’s it*” Girl 2 “*Me when I see someone who is drunk or something like that, I don’t like it. I mean… I don’t know. I wonder, “why doing that? What’s the point?” It’s like what do they gain out of doing things like that*”

On the other hand, tobacco products are considered as being far more dangerous than alcohol. Adolescents associate tobacco with cancer, disease and addiction. A minority of adolescents associated both tobacco and alcohol with disease. As with alcohol, adolescents think that people posting about themselves using tobacco products do it to make themselves look more mature and to impress their contacts. Adolescents report telling the ones they love that they should refrain from smoking or from having a behaviour that is bad for them. Yet, they also think that people should make their own choice when putting themselves at risk, and they would not go against their will.
FG 2Girl “*I think that it’s worst. I mean, it’s better to post alcohol than tobacco*” Boy 1 “*Yes exactly*” Boy 2 “*Tobacco causes damages that are irreversible, I think it’s a shame that… we have just one life; it’s a shame*” Boy 1 “*Too bad for them, I mean it’s their life, not mine*”FG 7Girl 1 “*I think that tobacco is more choking than alcohol because tobacco it’s really…*” Girl 2 “*Both are the same actually. For me, both…*” Girl 1 “*tobacco there are more risks associated to cancer or to really serious diseases, whereas not necessarily with alcohol. Because alcohol, nearly everybody drink some, whereas cigarette is much less common.*” Girl 2 “*for me it’s the same so…*”FG 8Boy: “*It’s negative, both of them [alcohol and tobacco]*” Girl: “*I think that the worst, in any case, it’s to smoke for sure. I mean, you inhale something that is bad in your lungs […] whereas alcohol, it can disappear over time. I mean it damages…*” Boy: “*nothing*” Girl: “*It damages your mind for a while, and then, after, it’s ok, you’re…*” Boy: “*Yeah but no*” Girl: “*It’s better than smoking, that damage your lungs*”

Adolescents do not mention following any brand linked to alcohol or tobacco products on SNSs. Adolescents report following brands associated with beauty products or clothes, or with accounts linked to their interests, among which celebrities could be a part. They mention product placement that celebrities integrate to their SNS accounts.
FG 3Girl 2 “*On the Instagram of Adidas for example, well, they show, every time there is something new that is out, they show it, so it’s interesting*” Girl 1 “*And for me, there is… my hobby is horseback riding so I always follow the Instagram account of horseback riding” […] and clothes, etc.*” Girl 3 “*I follow celebrities as well*” Girl 2 “*Me, I love woman YouTube influencers*” Girl 1 “*Yes me too*” Girl 3 “*Me it’s more people from TV series*”FG 6Interviewer “*What kind of brands?*” Girl 1 “*Hollister*” Girl 2 “*Adidas, Puma…*” Boy “*Brand of, It’s more clothes*” Girl 1 “*Nike*” Interviewer “*Clothes? Aren’t you following brands associated to… well we’ve talked about alcohol, tobacco, food…*” Girl 2 “*No, no I don’t even know if it exists*” Girl 1 “*Yeah… I never even tried to look for…*”

### 3.8. Influence Associated to SNS Use

Adolescents were asked to share their opinion about the possible influence of what they see on SNSs, and their own or others’ behaviour. Only a few groups reported the possibility of being influenced by SNSs. Adolescents acknowledged reproducing pictures that they found to be nice or to get inspired by some of the clothes that they might see through the posts on their feed. However, the majority do not think that they can be influenced by SNSs; they associate this risk with the naivety of people.
FG 8Interviewer “*Do you think that we can be influenced by SNSs or not?*” Girl 1 “*Yes*” Girl 2 “*Yes a lot*” Boy “*If you want to mess around, you have to look at what the others do. For example drinking, well, it will prompt you to be friends with them and they will drop you in*” Girl 2 “*You’re not wrong*” FG 3Girl 3 “*for example when we find a picture nice, we will try to do the same*” […] Girl 2 “*for example, YouTube influencers post pictures with a specific pose and if we find it nice, we will try to reproduce it, and if it’s nice, we will post it on Instagram*” Girl 1 “*It really depends on people, some do not care at all and will post whatever they want, other will pay more attention and will try to do the same as others*”FG 10Interviewer “*Do you think that what we see on the SNSs could influence us or not?*” Boy 1 “*Yes*” Girl “*Yes*” Boy 2 “ *It’s to make people envious*” Boy 3 “*Impressionable, I mean it depends on the people. Some are more easily influenced. As for me for example, I’m not influenced by people that… I don’t know, if I see people going on holidays I think “good for them, they are on holidays, it’s their business”. Me, I’m not influenced in the way that; sometimes, I am following some account with posts about clothes and I like them: well the ones that I like, I save them for later and then, one day, if I find them in a shop and all… I mean it’s only things like that*” 

As previously mentioned, the main risk associated with the use of SNSs remains for adolescents linked to their privacy. Adolescents are aware that their use of SNSs can lead them to have some problems, especially when it comes to sharing personal information, or when posting pictures of themselves.
FG 2Boy 1 “*There is also a bit of exposition, in the way that we get exposed to… well first to people that can see us and also to some danger I think*” Boy 2 “*like the hackers or paedophiles*”FG 6Boy “*You have to be careful because after the pictures that you post could be found everywhere on the web. Because if you do, you can be humiliated or even be harassed by people, like “you are ugly on this picture” for example and that they are saying it every time you post a picture. In every case, you have to be careful to what you post and the people you add to you Instagram account or every social network sites*”

The other risks reported to the interviewer by the sample are the existence or certain features associated with an SNS, which allows for anonymous commenting on their posts and content. These risks are linked to the lies they could be facing from their contacts and to online harassment.

Indeed, adolescents explain that people do not always tell the truth on SNS. Adolescents report that people can pretend to be someone they are not, and that they can be faking friendships online. This situation is reported as being common, especially with some online games that are played by adolescents. They mention as well the existence of applications that preserve anonymity and that are used by some malicious people to bully other people within that context.
FG 6Girl “*There is a type of social network site that is not really a social network site, actually it goes on the social network sites through a link, and it’s Sarahah and Askip Co (Askip has been turned down at the moment of this work has been written). It’s actually messages, but anonymous. It means we receive them and we post them on our story and we answer but we do not know who sent them*”FG 1Girl 1: “*I think that the people who do that are just…*” Girl 2 “*they are phony, they have no friends*” Group agree […] Girl 1 “*It knocks one down behind one’s back, it’s like “oh, I love you so much” and on and after you hear from your former best friend “well this one called you that name*” Girl 2 “*that is… it really bugs me*” Boy 1 “*Super…*” Boy 2 “*The joy of social networks*” Girl 2 “*And then it’s like “come in private when you want, I love you so much*” Boy 1 “*Oh well, it’s not all about happiness on social network you know. You can be harassed*” Girl 2 “*Yes, but that… yes… but it’s different*” Girl 1 “*Yeah, there are these messages like “you’re fat, you’re ugly… you have spots…*”

When facing these types of attacks, adolescents learn how to protect themselves by using the setting offered by the SNSs.
FG 6Girl 1 “*Let’s say that he doesn’t like me, I mean for example he comments “you’re ugly”, well, then there are 3 dots and I can click on them on choose “hide my picture” well for John for example*” Girl 2 “*We can block people, I mean we can block them or… how do we say?*” Girl 1 “*report*” Girl 2 “*report, it means for example if someone have attack you or is mean to you, well you can report her. It doesn’t change anything but… and you hide her from your feed*” Boy “*When you report someone, then Instagram they take it into account and they can ban this person from the social network site.*”

One boy mentions finding Snapchat “too addictive” and that SNSs are designed “to make money, to get people hooked on their phone” (FG 4). Yet, this point is not discussed further by him or the other members of the focus group, and neither by the other participants in the other focus groups.

Few adolescents talk about their parents and the control their parents have over the use they make of SNSs. A girl mentions having an account on Snapchat only and not Instagram, because of her mother, who said there was less risk associated with Snapchat. Other adolescents explain having been grounded by their parents from their SNS for reasons unrelated to their SNS use, or when risky behaviours associated with SNS were noticed by the parents.
FG 2Girl*[Answering why she has an account on Snapchat but not on Instagram] “Because my mother doesn’t want to and also because it’s less risky… that’s what she told me.*”FG 6Girl “*Me, one day, I’ve posted… I mean it was a guy, his name is, I don’t even remember who he was, but I knew who he was and I have received many messages so I knew it was him and my mother see it, that’s it. So I have uninstall everything*”

In a broader view, adolescents mention the need to be vigilant on the Internet and SNSs, that some information is distorted or fake. However, adolescents are not clear and precise about it, and some of them get confused with the information provided.
FG 2Girl “*there are things that are modified, there are things that… it’s not true like. It’s not really reliable. It’s not very accurate, well, it depends. […] Sometimes there are small logo, when there is a little tick within a circle, it’s official*” Boy “*yes it means it’s official. Https it’s official*” Girl “*I don’t know… I know that “dot gov” is like… it’s accurate things*”

### 3.9. Key Points from the Results

- Snapchat and Instagram are the two first SNSs that are the most used by adolescents from the focus groups at the time of the study (1st semester 2018).
- Snapchat is associated with friendship, spontaneity and privacy, whereas Instagram is associated with an official profile to be presented widely.
- Adolescents from the focus groups seem to make a balance between their face-to-face interaction with their friends and their online interactions with their contacts.
- The use of gimmicks in relation to friendship created by SNSs are questioned by adolescents, as they report both positive (e.g., stay in touch) and negative (e.g., grieving the loss of a symbol of friendship) outcomes from them.
- Adolescents start to elaborate on the possible influence of SNS content over their behaviour when referring to post being replicate (e.g., taking picture during holidays), or when wishing to buy clothes advertised (e.g., through sponsored post). Yet they do not make clear associations between SNSs content and health related behaviour.
- Although adolescents negatively judge their peers who are “showing-off” on SNSs using alcohol or tobacco products, they trivialised the presence of these products online.
- The presence of alcohol products within SNSs posts is commonly accepted by adolescents when used responsibly or during particular occasions (e.g., birthday), as being integrated to their regular life.
- Tobacco products are seeing as being deadly and far more dangerous than alcohol, which is mainly linked to party, food and friends before intoxication and hangover.

## 4. Discussion

In this explorative qualitative study, 39 French adolescents provided the researchers with information about their use of SNSs, about their perception of A+T products and they expressed their thoughts about the risk of influence they could encounter while using SNSs. This article gives a good insight into adolescents’ understanding and use of SNSs. The quotes reported can be associated with a life course perspective: adolescents’ perception of the values linked to A+T products is already influenced by external factors (e.g., family); still, they are at a time of their life when their own choice associated with the use of these substances is under consideration. Thus, preventive and health promotion strategies could be developed to counteract the influence of online unhealthy behaviours. Improvement of eHealth literacy could be an objective to reach [53].

### 4.1. Appraising the Influence of SNS Content and its Impact on Health

The adolescents interviewed seem to be down to earth when it comes to their SNS use, and do not seem to present any pathological use or consequences that could present any immediate risk to their health. However, adolescents do not seem to be fully aware of the risks linked to the use of SNS, particularly when it comes to the potential influences of the content of the SNSs’ posts on their own behaviour.

Adolescents acknowledge the existence of user-generated content linked to a brand, but they associate it with social influencers who are paid to integrate a brand to their own posts. When it comes to sponsored posts, adolescents do not give the same appreciation to the post as to regular ones. They would refrain from liking them and would refrain from commenting on them [54].

The adolescents who participated in the focus groups mentioned doing the same towards posts that stress a behaviour that is not acceptable or suitable for people their own age: they refrain from liking posts of their peers showing them smoking or drinking just to “show off”. According to the adolescents from our focus groups, they also do not seem to face much A+T paid advertising online, but could not guarantee it for sure. This uncertainty is worth noting, as adolescents do not seem to be aware of the laws in place regulating A+T advertisement (e.g., it is illegal to advertise tobacco products and the promotion of alcoholic beverages is restricted to certain conditions and totally forbidden towards minors) [55]. Moreover, adolescents usually tend to overestimate the number of people using substances, not only because a “deviant” behaviour is more noticeable. However, because the association of the substance to a positive behaviour increases the visibility of that substance while viewing [56,57]. In our case, the small amount of people using A+T substances reported by our respondents could be explained by the age of our sample when substance consumption is in its infancy and begins to develop from experimentation to misuse (e.g., alcohol intoxication) [11,12].

Whereas adolescents reported not validating their peers posting about themselves drinking or smoking, they were, at the same time, excusing these actions if taking place during particular occasions (e.g., family event, celebration). This perception associated with alcohol use, and misuse could be explained by the norms surrounding them within society, the family unit and also by the influence of messages integrated in traditional media [19,58,59,60]. It is the same for tobacco products: fewer people are smoking, which could explain the disagreement of adolescents towards the people who present themselves with a cigarette [13], no matter the context.

Adolescents are aware of marketing practices [18] and point out the partnerships that celebrities could establish with brands. Yet, some brand or product placement integrated within regular posts can be harder to notify. Indeed, as adolescents have a different use of SNS, so are the promotional messages and user-generated content that are displayed on these different media [61]. Specificities given to each SNSs need to be acknowledge in a public health perspective; whereas users would not have the same expectancies and intentions when connecting to different SNS, commercial strategies would adapt themselves to fit within these different uses.

Studies have shown that exposure to online behaviour is associated with decision making when it comes to adolescents’ risk behaviour [62]. According to our sample and without previously giving consideration to the presence of A+T substances online, they did not recall seeing many posts promoting A+T substances. Yet, after probing by the researcher, they actually reported seeing a good number of substances within the posts of their contacts. This should be monitored carefully and with details: more than the amount of exposure to which adolescents could be exposed, the connectivity towards the person posting with the substance has to be taken into account as the behaviour could be internalised by the viewer [63] and could have a strong impact [60].

Then, concern should be given to the use of SNS specificities towards people that are unstable or more fragile, or more generally towards adolescents who are in a period of their life when they are more vulnerable emotionally. As seen, values that are important for adolescents are at play within SNSs’ use (e.g., friendship), and gimmicks that are developed by the SNSs’ platforms based on these values (e.g., Snapstreak) to encourage adolescents to use their profile regularly should be questioned. Studies have documented such impacts and advocated for the need to allow adolescents to master the use of SNSs [2,5]. The adolescents from our focus groups were making some links between the influence of advertisement or product placement over their behaviour (e.g., buying clothes advertised), but did not seem to be fully aware of the many variables at stake regarding the influence of the content of media over their health. They were aware of existing dangers linked to use of the Internet; talking to strangers and posting pictures of their body as some rules that were learnt (e.g., through educational programmes at school). Yet, they did not mention possible influences on behaviour [1,62], mental health outcomes [64,65] or other factors.

### 4.2. SNS Understanding, Use and Expectations

In the literature, or when looking at the description of the applications that are associated with an SNS, the definition given to SNS remains unstable, and no common agreement within the scientific community allows us to describe precisely what an SNS is and what an SNS is not [66,67]. This is particularly true when looking at the evolution of the applications over time, adding specificities to their main content. Thus, it is not surprising that adolescents were not able to give a precise and clear definition of what an SNS is, although they pretended to know at first.

The main utilisation of SNSs reported by the adolescents from the focus groups did not differ from existing research. Adolescents report using SNS because they identified that most of their peers are also using SNS. Adolescents are as also likely to have more than one SNS account, using each SNS for a different purpose or to connect with different groups [68,69]. Adolescents are looking to stay connected to their friends, to their relatives and more widely to their contacts. These links could be acknowledged by the likes that are given more to the person, rather than being an appreciation of the content of the post itself.

They use SNSs to communicate and to share information with their friends and to stay informed about subjects that matter to them [70,71]. The sense of belonging or the need to bond with people that they have chosen to bond with, in a more or less distant way from their immediate family, is a common practice within human development and the construct of identity [22].

However, this multiplicity of the use of different SNSs seems to bring confusion to adolescents and to their understanding of each of the SNS functionality that can be set to protect oneself. As previously reported in the literature, adolescents seem to have a false sense of security, by overestimating the privacy settings they may have applied [1].

### 4.3. Future Directions and Recommendations

More research should be completed on the content shared and posted by adolescents. Studies have shown that receptivity to the marketing of substances is associated with a higher risk of substance use and intoxication [72]. Educational programmes aiming to empower adolescents should be developed with an aim to increase the adolescents’ awareness and identification of influential content embedded in SNSs and the harm that this content may have on them as individuals. These programmes need to pay particular attention to the environmental and cultural context to ensure maximum benefit [73].

It could be interesting to develop preventive programs that integrate SNSs within a health literacy perspective, so that adolescents can learn to protect themselves from the risks linked to their own use of SNS. Programmes aiming to empower adolescents towards a safe use of SNSs need to include “people knowledge, motivation and competences to access, understand, appraise, and apply health information in order to make judgments and take decisions in everyday life concerning healthcare, disease prevention and health promotion to maintain or improve quality of life during the life course” [53].

SNSs have transposed into their platforms classical games used by youth (e.g., truth or dare). Playing games is an important phase that is part of the developmental process and part of the identity construct [74,75]. Adolescents need to have their own experiences and to test their limits through more or less risky games [76]. Within this perspective, SNSs could be of interest, since the consequences of behaviours through the virtual reality could be tested, and the negative consequences affecting the real life setting learnt [77]. However, the specificities and the gimmicks that have been developed by SNSs to encourage adolescents to use their application on a regular basis (e.g., being on a Snapstreak) should be taken with caution, especially when they use the values and qualities of friendship that have been associated with either both engagement in problem behaviours and/or in positive development [62,78].

Developing a preventive program should not aim to tell adolescents what to do with their SNSs and how to use them precisely. Rather, they should support adolescents within their own use of SNSs and develop their capacity to judge the information provided within the content posted. Indeed, we have seen that SNS interactions and validation of posts shared online could be a way of showing care towards friends and standing up against risky behaviour in an unconventional preventive strategy [79]. Adolescents mentioned that they would advise their friends to refrain from undertaking risky behaviours towards their health e.g., smoking. However, they would also be at a place of acceptance, and they would not be judgmental towards their friends. Thus, adolescents could be empowered and encouraged to critically appraise posts from their friends, whether being for their own sake or for the sake of their friends. Encouraging adolescents to be critical towards the information they see on an everyday basis through their SNSs is important, so that they do not normalise online behaviours in a group size effect [80].

As well, social marketing strategies could be developed and integrated into SNSs used by adolescents, as another preventive strategy to improve adolescents’ eHealth literacy. As defined by the International Social Marketing Association and agreed by the social marketing community, “Social marketing seeks to develop and integrate marketing concepts with other approaches to influence behaviours that benefit individuals and communities for the greater social good” [81,82]. Developing such strategies could facilitate the motivation of the adolescents to resist the influence of the content posted on SNSs. Those strategies may take the form of preventive campaigns, based on the social marketing principles outlined above. They could use the same effective arguments that are employed by the industry to sell their products to adolescents, except that it would be for the greater good of adolescents’ health. Using social marketing strategies would involve the use of different steps in order to reach the objectives defined within a health perspective [82]. This study adds to the understanding of this adolescent population that may be used to inform such an approach.

Different strategies could be developed when it comes to preventing adolescents from being influenced by content spread on their SNSs. Adolescents are at a moment of their life where the information they receive from the world can be exaggerated, and where the weight of the consequences of actions on their health is underestimated, especially when it runs on the long term. It is then important not only to help adolescents to develop their eHealth literacy associated with SNSs, but also to facilitate their understanding of the risks at stake, by allowing a clear identification of marketing strategies.

It is important not to ignore the importance of SNSs and their potential impact on health when developing eHealth literacy strategies and programmes that aim to empower youth. The concepts of eHealth literacy and health literacy in a broader way are still evolving; whether being in their definition or whether being linked to the dimensions to take into account when operationalising the concepts [53].

### 4.4. Limitations of the Study

The study hereby presented includes a small sample of 39 adolescents. This number of participants is in line with this type of exploratory analysis but does not allow for generalisation or representativeness of the population studied. However, the data saturation point prior to terminating data collection has been reached, and still allows the researchers to compliment findings already published. As well, although a focus group technique might challenge some participants to consider different perspectives other than their own, it could also restrict other participants who are more reserved from expressing their opinion. In addition to this method, questionnaires could be developed and tested over a wider population. Further research might want to test empirically the phenomenon presented within this paper by analysing the user-generated content and/or the actual use adolescents have of SNSs.

This research has analysed the results all together for anonymous reasons, and has prevented the researchers in establishing difference between the communities interrogated. Future research with a larger and more diverse sample of the same age group might be considered, along with any differences within the context of socio-economics. It might be interesting, as well, to investigate further research towards an older population that are also in a vulnerable time of their life, and might not be aware of the influencing strategies at stake on SNSs: the young adults between the ages of 18–24.

A bias of social desirability has to be considered while considering the results published. As with any qualitative research using interviews or focus groups, a bias of social desirability may develop, which is as a tendency for participants to provide a response in line with what they believe to be socially acceptable. However, this bias might have been minimized, as researchers have used strategies to reduce it as much as possible (e.g., providing assurance to the participants, requesting examples, questioning indirectly).

As mentioned within the study, the SNSs’ specificities could change quite fast, and what might have existed at the time of the research might have evolved. As well, the authors would like to acknowledge that the information and specificities related to the SNSs presented are the ones mentioned by the adolescents interviewed at the time of the research. They do not pretend to define exactly the possibilities an SNS gives to its user, nor its limits, and do not claim to be accurate. The information provided within this study is that given by the adolescents, and they reflect their own understanding of the SNS that they discussed.

## 5. Conclusions

This study underlines the existence of the complex and extremely thin lines between the communication purpose of SNS, its entertaining side and the economic side associated with it by French adolescents. In line with previous published work [62], this study advocates for the need to develop efforts and programmes, aiming to educate adolescents on how to become health literate when it comes to the use of SNS. SNSs are part of the many functions of the Internet, and they facilitate the possibility to be connected to a vast number of people in all areas of the world. Overall, these focus groups demonstrate that adolescents are able “to access, understand and use information to promote and maintain good health” [83], however, their appreciation of what is health and the risk associated with SNSs’ use need to be supported. Educational programs should be developed and based on the functional, interactive and critical dimensions of the health literacy characteristics [83], while integrating adolescents’ existing competence and knowledge to maintain a high level of motivation to participate [53]. In addition, a social marketing approach should be considered within the perspective, to facilitate the understanding of the existing risks of influence on SNS, and to encourage the motivation of adolescents, to develop their competences to protect themselves from these risks.

## Figures and Tables

**Table 1:** Characteristics of the Focus Groups (FG) per setting, sex of the participants and duration.

	FG 1	FG 2	FG 3	FG 4	FG 5	FG 6	FG 7	FG 8	FG 9	FG 10	Total (*n*)
Setting (Yes = X/No = -)
Community centre	X	X	-	-	-	-	-	-	-	-	2
Private school	-	-	X	X	X	X	X	X	-	-	6
Public school	-	-	-	-	-	-	-	-	X	X	2
Total (*n*)											10
Sex (*n*)
Female	2	2	3	0	1	2	2	3	1	1	17
Male	4	3	0	5	4	1	0	1	1	3	22
Total (*n*)	6	5	3	5	5	3	2	4	2	4	39
Duration (h:min:s)
	01:10:15	00:49:19	00:48:25	00:37:08	00:58:59	00:45:34	00:31:06	00:43:57	00:35:15	00:38:40	07:38:38
